# Nucleic Acid Diversity in cGAS-STING Pathway Activation and Immune Dysregulation

**DOI:** 10.3390/biomedicines13092158

**Published:** 2025-09-04

**Authors:** Jingwei Guo, Mingjun Lu, Chenyang Wang, Dongchang Wang, Teng Ma

**Affiliations:** 1 Cancer Research Center, Beijing Chest Hospital, Beijing Tuberculosis and Thoracic Tumor Research Institute, Capital Medical University, Beijing 101149, China; 2 Department of Respiratory and Critical Care Medicine, Beijing Chest Hospital, Capital Medical University, Beijing 101199, China

**Keywords:** cGAS-STING, nucleic acid sensor, abnormal activation of cCAS-STING

## Abstract

The cGAS-STING pathway initiates the core cascade of innate immune defense by recognizing pathogen-associated and self-derived abnormal nucleic acids, and key molecules (such as cGAS, STING, downstream IFN-β, IL-6, etc.) may serve as biomarkers in various diseases. The diverse mechanisms by which distinct nucleic acids activate this pathway provide novel insights for therapeutic strategies targeting infectious diseases, cancer, and autoimmune disorders. To prevent aberrant cGAS-STING pathway activation, cells employ multiple regulatory mechanisms, including restricting self-DNA recognition and terminating downstream signaling. Strategies to mitigate pathological activation involve reducing nucleic acid accumulation through nuclease degradation (e.g., of mitochondrial DNA or neutrophil extracellular traps, NETs) or directly inhibiting cGAS or STING. This review elucidates the molecular mechanism of nucleic acid-mediated regulation of cGAS-STING and its role in disease regulation.

## 1. Introduction

Cells possess many sensors capable of detecting nucleic acids and are likely to have evolved to provide defense against viruses. For example, in the cGAS-STING pathway, dendritic cells, macrophages, and T cells usually highly express cGAS and STING, which are the main effector cells of the pathway [1,2]. Upon activation, these sensors initiate various signaling pathways within the cell, thereby stimulating the synthesis of type I interferons and other cytokines. This, in turn, stimulates immunological cells and enhances the immune status of the body. The cGAS-STING pathway is a significant signaling mechanism. Cytoplasmic cGAS directly recognizes exogenous (such as pathogens) or endogenous (such as mitochondrial/nuclear DNA) double-stranded DNA (dsDNA) and catalyzes the synthesis of the second messenger cyclic GMP-AMP (cGAMP) from ATP and GTP through conformational changes. cGAMP binds to STING protein on the endoplasmic reticulum (ER) and is transported to the Golgi apparatus via COPII vesicles. In the Golgi apparatus, STING oligomerizes and recruits phosphorylated TANK-binding kinase 1 (TBK1), thereby activating interferon regulatory factor 3 (IRF3). Phosphorylated IRF3 forms a dimer and translocates into the nucleus, initiating the transcription of type I interferon (IFN-I) and NF-κB-mediated inflammatory factors, driving anti-pathogenic or pro-inflammatory immune responses. Bacterial cyclic dinucleotides (CDNs) can also directly activate STING [3,4]. In recent years, an in-depth study of this signaling has shown considerable promise in terms of its potential applications across a range of fields, such as antiviral, antitumor, and autoimmune diseases. However, the activation mechanism, regulatory factors, and complex roles of this pathway in different physiological and pathological conditions remain unanswered questions that need to be further explored and elucidated to better utilize this key signaling pathway for disease treatment and immunomodulation.

**Figure 1:**
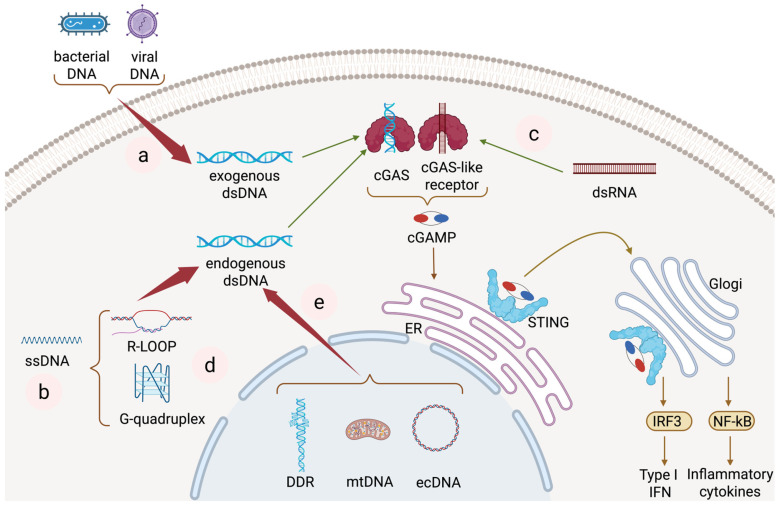
Activation of cGAS-STING pathway by different kinds of nucleic acid substances (graphic is created in BioRender. Guo, J. (2025) https://BioRender.com/tdrmn83). Schematic illustration of cGAS-STING pathway activation by diverse nucleic acids. The figure depicts how nucleic acid substances, including dsDNA, ssDNA, dsRNA, ssRNA, DNA-RNA hybrids, and extrachromosomal DNA (ecDNA), are sensed by cGAS, leading to cGAMP production and subsequent activation of the STING signaling pathway, ultimately inducing type I interferons and inflammatory cytokines. (**a**) dsDNA (viral/bacterial/mitochondrial DNA) binds to cGAS; (**b**) ssDNA activates cGAS through G-quadruplex/R-loop structure; (**c**) dsRNA is recognized by cGAS-like receptors; (**d**) DNA-RNA hybrids (such as R-loop) directly bind to cGAS; (**e**) extrachromosomal DNA (cedant) releases dsDNA fragments. cGAS synthesizes cGAMP → activates STING → induces type I interferon (IFN-I) and inflammatory cytokines. Created in .

Both human and mouse cells, including dendritic cells (DCs), macrophages, T cells, endothelial cells, and epithelial cells, function via the cGAS-STING pathway [1]. Continuous scientific explorations have gradually unveiled its mysterious veil. Ishikawa et al. (2008) initially identified STING as an endoplasmic reticulum junction protein involved in antiviral immune signal transduction in 2008 [5]. The team clarified the fundamental function of STING in DNA-mediated type I interferon signaling in 2009, establishing its role as a DNA immunosensor [6]. As a cytoplasmic DNA sensor identified in 2013, cGAS recognizes dsDNA in a sequence-independent manner but requires DNA length > 45 bp for optimal activation [7,8,9]. cGAS signaling functions intracellularly and plays a messenger role intercellularly [10,11,12]. The cGAS-STING pathway can be triggered not only by exogenous nucleic acids but also by the stimulation of its own genomic or mitochondrial DNA. The present study hypothesized that the dysregulated activation of cGAS-STING signal routing is involved in many autoinflammatory diseases, autoimmune diseases, and neurodegenerative diseases. Evidence supporting this hypothesis has been discussed in this study (Table 1) [13]. The predominant localization of cGAS is within the nucleus, regardless of the cell cycle phase [14]. A total of 85–95% of cGAS resides in the nucleus bound to chromatin, with mitotic release mechanisms remaining elusive [15]. cGAS activation was initially thought to originate exclusively from cytoplasmic activation, but its activation is also thought to originate in small parts of the nucleus; previous studies have shown the association between cGAS and condensed mitotic chromatin. Recently divided cells may retain cGAS in the nucleus and then redistribute it to the cytoplasm through mechanisms that remain unexplained [14,16]. A recent study highlighted how aberrant control of nuclear cGAS underlies the pathogenesis of interferonopathy Aicardi–Goutières syndrome. Specifically, Uggenti et al. found that loss-of-function mutations in *LSM11* and *RNU7-1*, two genes involved in histone precursor mRNA processing, lead to a disturbance in linker histone stoichiometry, which is associated with an increased cGAS dependence of type I interferon gene expression in patient cells [16]. The core mechanism involves cGAS sensing cytoplasmic dsDNA and producing the second messenger cGAMP. cGMP, cAMP synthase, and cGAS can identify dsDNA in the cytoplasm, including nucleic acid substances emitted by bacteria, viruses, or injured cells. Upon encountering the sugar-phosphate backbone, cGAS bonds and structural alterations initiate the synthesis of cyclic GMP-AMP (cGAMP) from ATP and GTP. cGAMP, produced by cGAS, operates as an immunotransmitter that regulates innate immunity from a distance. cGAMP is exported from and imported into the cell, facilitating cGAS-STING signaling among cells [15]. Moreover, neutrophil extracellular traps (NETs) serve as substantial reservoirs of extracellular genomic DNA and have demonstrated the capacity to stimulate cGAS upon phagocytic absorption [17]. cGAMP swiftly disseminates within the cell and is associated with the transmembrane protein IFN Gene Stimulating Factor (STING) located in the endoplasmic reticulum (ER) [18,19]. Diffusible cyclic dinucleotide cGAMP can be passed from cell to cell, thereby realizing a variety of cGAMP-mediated actions within cells of the adjacent tumor microenvironment [20,21]. Following cGAMP binding, STING undergoes trafficking and conformational changes to initiate signaling. Substantial conformational alterations in STING initiate its relocation from the endoplasmic reticulum to the ER-Golgi intermediate compartment (ERGIC) and Golgi apparatus, a mechanism reliant on the cytoplasmic coat protein complex II (COP-II) and ADP-ribosylation factor (ARF) GTPase [22,23]. However, the mechanism by which STING is transported is not yet well understood. So far, research has shown that not only dsDNA can activate the cGAS-STING pathway; dsRNA and DNA-RNA hybrids can do so as well [24]. Over the course of evolution, the ligand specificities and cGAS-like receptors in fruit flies of the genus Drosophila are specific for dsRNA and not dsDNA [25,26,27]. STING activation ultimately leads to the transcription of type I interferons and inflammatory cytokines. Type I interferons, pro-inflammatory cytokines, and chemokines are upregulated because upstream IRF3 and nuclear factor kappa B are activated by STING protein activation [28]. The C-terminal tail (CTT) of STING protein first recruits TBK1 through a conserved PLPLRT/SD amino acid binding motif, which promotes dimerization-mediated autophosphorylation of TBK1 [29,30]. In turn, the activated TBK1 phosphorylates STING at Ser366 in the CTT. The CTT is part of the pLxIS motif (where p represents a hydrophilic residue, x represents any residue, and S represents a phosphorylation site) and allows the STING-TBK1 complex to recruit IRF3 [31], which is then phosphorylated by TBK1, leading to its dimerization, nuclear translocation, and target gene induction. The activation of STING can also activate inhibitors of the nuclear factor-kappa B (NF-κB) kinase (IKK) complex, thereby promoting the activation of NF-κB transcription factors. NF-κB translocates to the nucleus, where it promotes the transcription of inflammatory cytokines, tumor necrosis factor (TNF), IL-6, and IL-1β [13]. After STING activation, it buds from the endoplasmic reticulum (ER) membrane via the COPII vesicle, forming a transport vesicle. The COPII vesicle transports STING from the ER to the ER-Golgi intermediate compartment (ERGIC). In the ERGIC, STING is handed over to the COPI vesicle and eventually reaches the Golgi apparatus [28]. Although COPII vesicle transport is widely accepted, recent studies suggest that there may be a STING activation pathway independent of the Golgi apparatus under cellular stress conditions. We need to continue exploring this area of research [32]. Additionally, STING stimulation has been demonstrated to independently induce autophagy and cell death reactions, in addition to activating downstream transcriptional programs [20,27,28]. In tumor cells, STING-dependent IRF3 activation is crucial for generating novel antitumor CD8 T cell responses, thus integrating intrinsic and adaptive immunity [29]. STING in tumor cells aids in the tumor rejection response in prostate cancer; however, its activity is frequently inhibited in these cells, partly through the JAK2 and STAT3 pathways [33]. As shown in Figure 1, various nucleic acids activate this pathway through different molecular mechanisms.

## 2. dsDNA in cGAS-STING Activation

Double-stranded DNA (dsDNA) is the primary heritage material in living organisms. There is a wide range of dsDNA sources, including exogenous sources (viruses and bacteria) and endogenous sources (mitochondrial DNA and nuclear DNA leakage). Mitochondrial DNA, viral DNA, bacterial DNA, and free double-stranded DNA to the environment can all trigger the cGAS-STING pathway [9,52,53,54]. cGAS recognizes dsDNA in a manner that is independent of sequence [9]. The C-terminal nucleotide transferase domain of cGAS contains three positively charged DNA-binding sites: Site A consists of residues K347, K394, etc., which directly bind to the sugar phosphate backbone of dsDNA. Its function induces conformational changes in cGAS, opens the catalytic pocket, and allows substrates (ATP/GTP) to enter the catalytic site. Site B is composed of residues such as R236 and K335 and is adjacent to site A. Its function is to stabilize the cGAS dsDNA complex (the smallest functional unit) and promote dimerization. The third site (non-essential) serves to assist in the extension binding of long dsDNA. When the length of dsDNA is less than 20 bp, dsDNA only binds to site A and cannot alter the cGAS conformation, with only weak activation or no activity. When the length of dsDNA is greater than 45 bp, dsDNA occupies both sites A and B, triggering dimerization and subsequent cascades (shown in Figure 1a) [55,56]. cGAS, which has been activated by dsDNA, is responsible for catalyzing the synthesis of the second messenger 2′,3′-cGAMP from ATP and GTP. The messenger is responsible for binding and activating STING [38]. As the first signaling molecule discovered to activate the cGAS-STING pathway, triggered by dsDNA, it plays a significant role in the progression of many diseases. Self-DNA, including nucleosomes and mitochondrial DNA, stimulates the STING cascade through cGAS, driving SLE-like autoimmune responses. In animal models, knocking out the cGAS gene significantly decreases the autoimmune phenotype (Table 1) [45]. It has also been proven that stimulation of the cGAS-STING signaling pathway is triggered by the abnormal accumulation of dsDNA in the cytoplasm and occurs in a number of disorders, such as amyotrophic lateral sclerosis, newborn hypoxic–ischemic encephalopathy, and cerebral ischemia/reperfusion injury (Table 1) [50,57,58]. A recent study discovered that cGAS can bind to CDNs [59]. CGAS detects double-stranded DNA (dsDNA) from various sources, exogenous dsDNA, such as invading viruses and bacteria, and endogenous dsDNA generated by self-damage, establishing a robust molecular foundation for the diverse actions of cGAS in the innate immune system.

Long-chain exogenous pathogen DNA has high activation efficiency, directly inducing cGAS phase separation and explosive IFN-I response, and can clear infection in the HSV-1 model. The self-DNA of extracellular sources has chronic weak activation properties. Apoptosis/NETs DNA breaks through the DNase II/LAP degradation barrier and activates cGAS, driving autoimmunity such as SLE. The mitochondrial DNA released by stress has a sustained activation effect on the cGAS STING pathway, and mitochondrial damage (BAX/VDAC pore) releases oxidized mtDNA, mediating neurodegenerative diseases such as Parkinson’s disease. After the barrier is breached, the nuclear DNA escapes the intracellular inhibition (nucleosome) through micronuclei/retrotransposons, leading to pathological activation of the cGAS STING pathway and resulting in aging and genomic instability (Table 1) [28]. Alternatively, the presence of mitochondrial DNA or anomalous telomere-derived DNA in the tumor microenvironment may act as an external stimulus for cGAS in the phagocytic cells that comprise the tumor bed (Table 1) [39,60]. The emergence of nuclear-like structures in the cytosol, termed MN (a form of chromosome abnormality is micronuclei (MN), which arise from chromosomes or chromosome fragments that fail to incorporate into daughter nuclei during telophase and recruit their own defective nuclear envelope), can result from chromosomal instability. This instability may result from genomic lesions that remain unrepaired or deficiencies in the precise segregation of chromosomes [61,62]. The mitotic progression of cells after genotoxic cancer treatment leads to the formation of MN, which exhibits interferon-stimulated gene (ISG) characteristics dependent on the cGAS-STING pathway [63]. In contrast to the direct activating effect of dsDNA, DNA-binding proteins, especially ZDNA-binding proteins, also contribute to the activation of the cGAS-STING pathway. Z-DNA-binding protein 1 (ZBP1), also known as the DNA-dependent interferon regulator activator (DAI), is another cell membrane NA sensor expressed in human B lymphocytes, mouse fibroblasts, and T cells [64,65]. The phenomenon under investigation has been shown to stimulate the signaling pathways of TBK1, IRF3, IFN I, and NF-κB, promote inflammasome activation, and trigger apoptosis in PAN cells [66].

## 3. ssDNA in cGAS-STING Activation

While dsDNA is a primary activator, single-stranded DNA (ssDNA) can also engage the cGAS-STING pathway through specific mechanisms. Single-stranded DNA (ssDNA) activates the MyD88 pathway and induces type I interferon and inflammatory factors via TLR9 [67]. ssDNA activates the cGAS-STING pathway by forming secondary structures (e.g., hairpins and G-quadruplexes) that mimic dsDNA. In aging microglia, the G4 structural stability of ssDNA or mRNA increases, leading to overexpression and sustained activation of the STING protein (Table 1) [51]. ssDNA can also bind to host proteins, such as RPA, triggering cGAS recognition and the activation of downstream proteins in this signaling cascade [68]. SsDNA caused by replication stress or chemotherapy can activate stimulation of the cGAS-STING regulatory pathway. PARP inhibitor therapy reduces ssDNA breaks and has pathological significance, especially in deficient cells [69]. During infection or acute DNA sensing, STING promotes inflammation by activating the TBK1-IRF3 pathway, driving the production of type I interferons (IFN-α/β) and pro-inflammatory cytokines (e.g., TNF-α, IL-6) to combat pathogens. Under chronic settings like autoimmunity, STING enforces immunosuppression by constitutively maintaining the expression of key immunoregulatory genes (*A20/TNFAIP3*, *SOCS1*, *SOCS3*, *IDO-1*), which restrain TLR signaling, limit myeloid hyperactivation, and promote regulatory T cell (Treg) function to prevent pathological inflammation [70].

## 4. Double-Stranded RNA in cGAS-STING Activation

Expanding beyond DNA ligands, double-stranded RNA (dsRNA) has also been identified as an activator of the cGAS-STING pathway. Double-stranded RNA (dsRNA) mimics the structure of viral dsRNA and serves as a pathogen-associated molecular pattern (PAMP) recognized by intracellular pattern-recognition receptors (PRRs) [71]. Most cancer cells detect cytoplasmic RNA and activate the RIG-I–MAVS–IRF3 signaling cascade, inducing IFN-β secretion. In contrast, human cancer cell lines co-expressing functional cGAS and STING exhibit activation of the cGAS-STING pathway (evidenced by TBK1/IRF3 phosphorylation and ISG expression) upon cytosolic DNA sensing yet fail to secrete IFN-β [72]. Previous studies have indicated that cGAS-like receptors in Drosophila can recognize dsRNA. The cGLR1 of fruit flies (encoded by gene CG12970) acts as a cGAS-like receptor, directly binding to long dsRNA (>30 bp). The ligand binding surface of cGLR1 has undergone evolutionary remodeling (compared to human cGAS, it lacks the Zn ribbon motif), allowing it to specifically recognize RNA rather than DNA. DsRNA binding activates cGLR1, catalyzing the synthesis of non-classical cyclic dinucleotide: 3′,2′-cGAMP. 3′,2′-cGAMP can resist degradation by viral poxin nuclease (poxin can cleave 2′,3′-cGAMP), enhancing antiviral efficacy. The fruit fly STING (dSTING) specifically binds to 3′,2′-cGAMP (almost does not respond to 2′,3′-cGAMP). Researchers have discovered for the first time that 3′,2′-cGAMP acts as an endogenous STING agonist, expanding the diversity of cyclic dinucleotide signaling (shown as Figure 1c) [25]. We also noted that RNA-binding proteins facilitate the triggering of the cGAS-STING pathway, regardless of the potential RNA-binding capability of cGAS, which may play an important role in the natural immune antiviral response [73]. However, it is important to note that the RNA-binding activity of cGAS is distinct from that of other RNA sensors, including RIG-1 and TLR3, which are primarily capable of recognizing viral RNA [74].

## 5. Single-Stranded RNA in cGAS-STING Activation

Single-stranded RNA (ssRNA) is an important class of biomolecules that play a significant role in the onset, progression, and supervision of cancer. RNA strands in RNA-DNA hybrids (such as R-loops) can activate cGAS [75]. ssRNA can initiate the cGAS-STING pathway by assembling into r-loops and G-quadruplexes (G-quadruplex is a quadruple-stranded structure formed by nucleic acid sequences rich in guanine, which is involved in gene expression regulation and immune signal activation). ssRNA does not directly activate cGAS (unlike viral DNA) but optimizes the activation and aggregation of cGAS in a low-DNA environment, thereby promoting the antiviral response of the STING pathway. However, at high DNA concentrations, it competitively inhibits cGAS. In the cytoplasm, when the concentration of dsDNA is low, tRNA binds to cGAS to form liquid–liquid phase separation condensates. These condensates serve as platforms to concentrate and stabilize cGAS, promoting its binding to a small amount of dsDNA, enhancing cGAS dimerization and catalytic activity, and thereby activating the STING pathway [71,76].

## 6. DNA-RNA Hybrid in cGAS-STING Activation

Recent research has shown that DNA-RNA hybrids can stimulate the cGAS-STING pathway under certain circumstances. The cGAS protein directly binds to RNA: DNA heterodimers and synthesizes cGAMP (shown as Figure 1d) [75]. In R-LOOP, DNA and RNA assemble into a triplet structure, and transcriptional stress or defects in RNA helicases (e.g., DHX9) result in the accumulation of R-loops, whose exposed single DNA strands are recognized by cGAS instead of the traditionally perceived dsDNA [24]. The RNA strand is removed by RNase H digestion, and the remaining DNA single strand still activates IFN-β secretion (cGAS) [75].

## 7. Extrachromosomal DNA in cGAS-STING Activation

Extrachromosomal DNA (ecDNA) is a circular DNA molecule located in the cell nucleus that operates independently of chromosomes. They usually carry key oncogenes and their regulatory elements (such as promoters and enhancers), which can be inherited through random allocation in cancer cells [77]. Owing to the absence of centromeres in ecDNA, it is unevenly inherited by daughter cells during cell division, resulting in a high degree of variation in the gene copy number. In 1965, researchers first detected ecDNA in neoplastic cells known as “minute chromatin bodies”. This discovery marked the beginning of ecDNA research, but little is known about its function and mechanism [78]. During the 1980s, numerous transmission electron microscopy (TEM) investigations indicated that ecDNA was circular and comprised chromosomal fibers carrying nucleosomes [79,80,81]. In 2017, Mischel et al. analyzed 17 types of human cancers and found that ecDNA was present in nearly 50% of tumors, whereas it was almost absent in normal cells. This study confirms the widespread distribution of ecDNA in various cancers and its important role in tumor evolution [82]. In 2019, Sihan et al. first directly analyzed the structure of ecDNA, elucidated its basic functions, and revealed the mechanism by which ecDNA promotes oncogene expression and tumor evolution through high transcriptional activity [83]. Cancer often manifests as amplification of oncogenes on extrachromosomal DNA (ecDNA) [82,84]. Pan-cancer research indicates that the oncogene encoded by ecDNA is among the most highly expressed genes in the tumor transcriptome, correlating elevated copy numbers with increased transcription levels [83]. These approaches may culminate in the liberation of substantial quantities of anomalous DNA pieces towards the cytoplasm that are acknowledged by cGAS signaling, thus activating the cGAS-STING pathway (Table 1) [41]. In 2021, Fitzgerald et al. discovered that ecDNA is not only present in advanced tumors but is also detected in precancerous cells such as Barrett’s esophagus, indicating that ecDNA contributes to the initial phases of tumor transformation [85]. ecDNA has now been detected in the majority of human cancer types, including glioblastoma, neuroblastoma, sarcoma, and medulloblastoma, consisting of head and neck, lung, esophagus, gastric, pancreatic, hepatic, colorectal, bladder, breast, ovarian, and prostate cancers [82,86]. The replication dynamics and chromatin structure of ecDNA further contribute to its immunogenic potential. EcDNA contributes to the initiation and progression of cancer through multiple mechanisms, such as enhancing oncogene activation via increased copy number or chromosomal contacts and acting as a reservoir for DNA recombination by reintegrating into and cleaving off chromosomes. Extracellular DNA (ecDNA) is a significant element that results in treatment resistance and an unfavorable prognosis in patients with cancer [84,87]. EcDNA can include oncogenes similar to those located on chromosomes [83,88], yet it follows a completely different inheritance mechanism [89,90]. EcDNA copies themselves during the S phase when they are in sync in conjunction with the remainder of the genome. Euchromatin usually undergoes replication prior to heterochromatin formation. Because of this and the fact that ecDNA has a more open structure, it mostly copies itself during the middle and early S phases rather than the late replication phase. Moreover, ecDNA replicates solely once during the complete cell cycle [91,92]. The circular structure of ecDNA makes it more accessible to regulatory elements such as transcription factors and enhancers in the nucleus, thereby increasing the expression level of oncogenes. Elevated expression may lead to the dissemination of many aberrant DNA fragments to the cytoplasm, which are then identified by cGAS, thereby activating the cGAS-STING pathway [41,42]. EcDNA usually has a more open chromatin structure, making it more susceptible to transcription and replication, which in turn increases the chances of DNA damage and fragmentation. The double-stranded DNA fragments released from ecDNA can also activate cGAS and trigger the cGAS-STING pathway (shown as Figure 1e) [41,43]. The circular topology and high transcriptional activity of ecDNA promote cGAS binding and the release of dsDNA fragments [44]. Through the indirect action of dsDNA, ecDNA can initiate a signaling cascade.

## 8. Diseases Associated with cGAS-STING Malfunction

**Figure 2:**
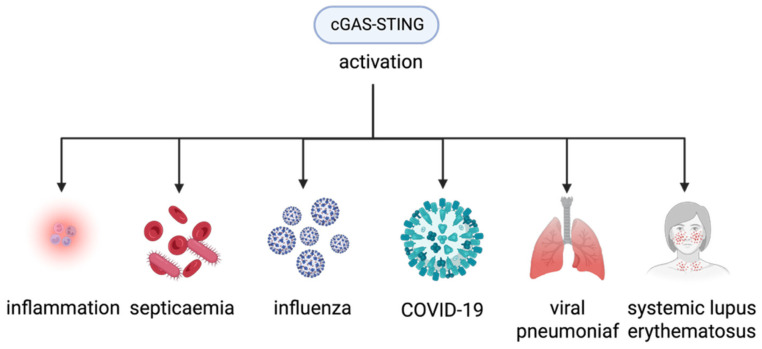
Aberrant expression of cGAS-STING in various disease conditions (graphic created in BioRender. Guo, J. (2025) https://BioRender.com/y54mmj8). Schematic illustration of diseases caused by aberrant activation of the cGAS-STING pathway. The figure summarizes various diseases associated with dysregulated cGAS-STING signaling, including infectious diseases and autoimmune diseases.

After significant DNA damage, which is a component of an inherent oncogenic mechanism or provoked by genotoxic therapy (for example, radiation or chemotherapy), some enzymes that break down DNA damage products produce dsDNA fragments that are not supposed to be present. These fragments cause cancer cells to produce an IFN-I signal that depends on cGAS (Table 1) [34,35,36,37]. Prolonged stimulation of the cGAS-STING pathway results in the persistent initiation of intrinsic immunity, which leads to inflammatory disorders. During cellular senescence, cumulative injury to nuclear and mitochondrial DNA triggers cGAS and induces senescence and systemic inflammation. They are widely considered abnormal occurrences that diminish longevity [15]. In the case of cancer, prolonged stimulation of cGAS-STING can also allow immune escape, thereby diminishing the effectiveness of immunotherapy or co-enhancing tumor function (Table 1) [38]. Deviant stimulation of the axis results in the emergence of infectious illnesses, such as bacterial infections (e.g., sepsis), viral infections (e.g., influenza, COVID-19), and autoimmune diseases, such as systemic lupus erythematosus (SLE), rheumatoid arthritis (RA), and other diseases (Table 1) [47,48,49]. Figure 2 illustrates the three major disease lineages caused by abnormal activation of this pathway. In viral pneumonia, mtDNA is released into the cytoplasm of the macrophages. This induces unexpected responses and exacerbates and exacerbates inflammatory reaction [93]. Consequently, it is imperative to safeguard cells from unforeseen and unregulated activation of innate immunity and eradicate the aberrant stimulation of cGAS-STING by nucleic acids.

**Figure 3:**
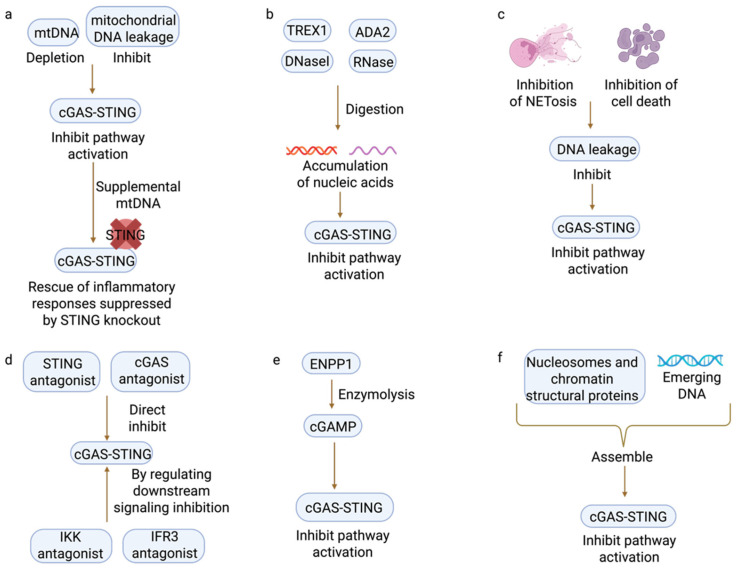
Mode of inhibition of cGAS-STING pathway activation (graphic created in BioRender. Guo, J. (2025) https://BioRender.com/v82sa06). (**a**) Depletion of medina and inhibition of mitochondrial DNA leakage. (**b**) Intracellular and extracellular DNase and RNase digest nucleic acids. (**c**) Inhibition of NETosis and cell death. (**d**) Various antagonists inhibit cGAS-STING. (**e**) ENPP1 eliminates extra-cytoplasmic cGAMP. (**f**) DNA and ribosomal and chromatin structural protein assembly prevent cGAS-STING binding activation.

Numerous mechanisms have evolved to safeguard cells against unforeseen innate immune stimulations. These mechanisms serve to either restrict the recognition of upstream self-DNA or terminate downstream signals. To prevent cGAS-dependent self-reactivity, the continuous elimination of self-DNA is crucial. This is accomplished by cytoplasmic 3′ repair exonuclease 1 (TREX1), lysosomal DNase II, or adenosine deaminase 2 (ADA2), both within and outside the cell (Figure 3b) [45,94,95]. Similarly, the synchronized function of nucleosomes and chromatin structural proteins, including self-binding barrier factor 1, inhibits the induction by intact genomic DNA (Figure 3f). The regulation of cGAMP is attained through the activity of the ATP-dependent transporter channel member ABCC1, and more transporters remain to be delineated. Collectively, these transporters restricted intracellular STING-mediated activation. Upon exiting the cell, cGAMP is destroyed by phosphodiesterase ENPP1. ENPP1 is a membrane-anchored protein featuring a catalytically active structural region that extends into the extracellular environment (Figure 3e) [96]. Along the signaling pathway, stimulation and modulation of STING are intricately associated with intracellular trafficking. Activated STING relocates from its homeostatic position in the endoplasmic reticulum, passing through the Golgi apparatus, the site of signal transduction, to the lysosome. Here, activated STING is effectively degraded [97,98].

In addition to preventing the abnormal activation of the cGAS-STING pathway, our body also has mechanisms to eliminate the effects brought about by the abnormally activated cGAS-STING pathway. At the nucleic acid level, the accumulation of various types of nucleic acids in cells can be eliminated using appropriate nucleases, such as DNase I and RNase. In terms of nucleic acid release inhibition, this can be achieved by the inhibition of mitochondrial DNA leakage, the loss of mtDNA impeding the cGAS-STING pathway, and the nuclear translocation of p65 and IRF3. In addition, mtDNA supplementation rescued the inflammatory response inhibited by STING knockdown. The experiment was conducted in the mDPC6T cell model system cultured in vitro. By simultaneously knocking down the STING gene and supplementing exogenous mtDNA in this cell line, it was demonstrated that cytoplasmic mtDNA drives the inflammatory response of odontoblasts by activating the cGAS-STING pathway (Figure 3a) [99]. Another way to inhibit nucleic acid release is by inhibiting NETosis and cell death. NETosis (neutrophil extracellular trap formation) is a specific form of programmed cell death that is actively triggered by activated neutrophils; it can lead to cGAS-dependent neutrophilic lung inflammation and ARDS (Figure 3c) [46]. This can be achieved by the direct inhibition of cGAS or STING (e.g., STING antagonists and cGAS inhibitors) or by modulation of downstream signaling pathways (e.g., IKK inhibitors and IRF3 inhibitors) (Figure 3d). Triggering the cGAS-STING pathway holds potential for cancer therapy. Nevertheless, prolonged STING triggers uncontrolled chronic inflammation, potentially culminating in extensive autoinflammation and autoimmune disorders. Chronic activation of cGAS-STING by auto-DNA may lead to autoimmunity. In addition, cGAMP transporters are also highly associated with autoimmune diseases. Sustained cGAMP proliferation and STING activation may lead to excessive IFN-I production or autoimmune responses in genetically susceptible individuals [13]. A variety of autoimmune diseases, including STING-associated vasculopathy in neonates (SAVI) and inflammatory bowel disease (IBD), have been associated with hyperactivation of the stimulator of interferon genes (*STING*) pathway [100]. It is crucial to rationally develop inhibitors that effectively target STING because STING inhibitors play a significant role in the treatment of autoimmune and inflammatory diseases. Contemporary STING inhibitors can be categorized into two primary kinds. The initial category comprises inhibitors that covalently attach to STING amino acid residues (e.g., Cys88, Cys91, and Cys148), thereby obstructing STING oligomerization and activation. The alternative targets the LBD at the C-terminus of STING and competitively obstructs the binding of endogenous ligands [101]. However, other studies have focused on the degradation of small molecules by STING proteins via ubiquitinated protein systems. The oligomerization of higher-order and post-translational changes (such as ubiquitination, phosphorylation, and palmitoylation) of STING is crucial for its control and may serve as significant targets for the creation of STING inhibitors [29,102,103,104,105].

## 9. Discussion

In summary, this review has highlighted that dsDNA is the core activator (with the highest efficiency when >45 bp), while ssDNA, RNA, and hybrids require indirect mechanisms such as structural mimicry (G-quadruplex) or formation of R-loop to activate the pathway. Although nuclear cGAS accounts for 85–95% of the total, its actual contribution may be underestimated due to its tight binding with chromatin; recent studies have revealed that dysregulation of nuclear cGAS directly leads to Aicardi–Goutières syndrome, challenging the traditional understanding that “cGAS is only activated in the cytoplasm”. The circular topology and high transcriptional activity of ecDNA significantly increase dsDNA fragment leakage, providing a new explanation for tumor immune escape and treatment resistance. However, there are still some contradictions in current research. Drosophila cGAS-like receptors specifically recognize dsRNA, while mammalian cGAS has been shown to bind to DNA-RNA hybrids. This evolutionary divergence suggests that the current sequence-based classification of nucleic acids may not be sufficient to explain the nature of activation, and secondary structure (such as Y-shaped DNA) or spatial conformation may be more decisive factors. Further research is needed to fully elucidate the direct role played by nuclear cGAS in DNA damage repair; the existing evidence only suggests its association with mitotic chromatin. Developing tissue-specific STING modulators, current inhibitors often target cysteine residues or ubiquitination modifications, but systemic inhibition may exacerbate the risk of infection. The recent co-crystal structures—especially the cGAS–dsDNA and STING–c-di-GMP complexes—now enable structure-guided fragment growing and covalent tethering strategies that could yield isoform-specific STING binders, thereby circumventing the cardiovascular liabilities seen with pan-agonists.

A remaining controversy is whether persistent low-level STING activation in tumors is immunostimulatory or immunosuppressive. Single-cell RNA-seq studies reveal that intratumoral dendritic cells can be “hyper-activated” yet functionally paralyzed, suggesting that timing and dosing of STING agonists may need to be synchronized with epigenetic checkpoint modulators (e.g., HDAC3 inhibitors) to restore antigen presentation. Conversely, in autoimmune models, germline gain-of-function STING alleles produce a feed-forward loop of mitochondrial DNA release, implying that organelle-directed antioxidants (e.g., mitoQ) might synergize with cGAS enzymatic blockers. Furthermore, nucleus architecture abnormality, including micronuclei rupture, nuclear envelope blebs, and chromatin herniations, that convert genomic instability into innate immune activation, strongly supports integrating DNA-repair modulators (PARP inhibitors, ATR inhibitors) with STING-targeted therapies, particularly in BRCA-deficient cancers where synthetic lethality can be extended to immune-mediated tumor clearance.

Moreover, the cGAS-STING pathway also shows great promise for application as a biomarker in various diseases. In infectious diseases, elevated expression or activity of cGAS-STING pathway molecules may indicate pathogen invasion (e.g., viruses, bacteria), aiding diagnosis of infection type and severity. In tumors, the activation-associated molecules serve as markers of tumor immune responses; high expression often suggests a better immunotherapy response and may monitor tumor progression or recurrence. In autoimmune disorders (such as systemic lupus erythematosus) and inflammatory diseases, molecular markers of pathway dysregulation (e.g., circulating STING protein, IFN-associated cytokines) reflect disease activity, aiding diagnosis and treatment evaluation. Furthermore, in neurodegenerative diseases (e.g., Alzheimer’s disease) and cardiovascular disorders (e.g., atherosclerosis), molecules within this pathway may serve as potential markers of disease progression, offering insights for mechanistic research and clinical management.

In conclusion, the cGAS-STING pathway is a key component of the innate immune system, with a complex molecular mechanism of activation by diverse nucleic acids. Its dysregulation is associated with a variety of diseases, and understanding its regulatory mechanisms and developing targeted therapies hold great potential for the treatment of these diseases. Future research should focus on further elucidating the molecular details of the pathway, identifying novel regulatory mechanisms, and translating these findings into effective clinical therapies.

## Figures and Tables

**Table 1:** Summary of cGAS-STING pathway roles in major disease categories.

Disease Category	Key Mechanisms
Cancer	Genotoxic therapies produce dsDNA fragments, activating cGAS-dependent IFN-I signaling [34,35,36,37].
	Chronic activation promotes immune escape and reduces efficacy of immunotherapy [38].
	Mitochondrial DNA or telomeric DNA in tumor microenvironment activates cGAS [39,40].
	ecDNA releases dsDNA fragments that activate cGAS-STING [41,42,43,44].
Autoimmune Diseases	Self-DNA (e.g., nucleosomes, NETs) aberrantly activates cGAS-STING, driving SLE-like autoimmunity [45].
	Chronic auto-DNA activation leads to autoimmunity (e.g., SAVI, IBD) [13,46].
Infectious Diseases	Bacterial infections cause inflammatory storm via aberrant activation [47].
	Viral infections (e.g., influenza, COVID-19): mtDNA release exacerbates inflammation [48,49].
Neurodegenerative Diseases	Mitochondrial DNA leakage activates cGAS-STING, mediating Parkinson’s disease [28,50].
	TDP-43 triggers mtDNA release, activating cGAS-STING in ALS [50].
Cellular Senescence	Cumulative nuclear and mitochondrial DNA damage activates *cGAS*, inducing senescence and systemic inflammation [51].

## Data Availability

Not applicable.
